# Exploratory Machine Learning Prioritizes Shared Blood Transcriptional Candidate Genes and Immune Correlates Across Antiphospholipid Syndrome and Systemic Sclerosis

**DOI:** 10.1155/bmri/8727103

**Published:** 2026-09-28

**Authors:** Renju Yang, Jinchan Liu, Yawen Liu, Fei He

**Affiliations:** ^1^ Dali University, Dali, Yunnan, China, dali.edu.cn

**Keywords:** antiphospholipid syndrome, immune infiltration, machine learning, shared candidate genes, systemic sclerosis

## Abstract

Antiphospholipid syndrome (APS) and systemic sclerosis (SSc) are distinct autoimmune diseases, but both are characterized by immune dysregulation, persistent inflammation, and vascular injury. To explore whether they also share blood transcriptional features, we analyzed peripheral blood datasets from GEO (GSE102215, APS: nine patients/nine controls; GSE231691, SSc: 49 patients/18 controls). Differential expression analysis performed separately in each cohort using edgeR with cohort‐specific fold‐change thresholds and nominal *p* < 0.05 identified 281 genes that were altered in the same direction in both diseases, including 100 upregulated and 181 downregulated genes. Enrichment analyses in both cohorts consistently pointed to interferon‐related, cytokine, and inflammatory pathways. We then combined random forest and a single‐hidden‐layer artificial neural network as exploratory feature‐prioritization approaches and identified five shared candidate genes: S100A8, IER5L‐AS1, LTK, PRR5‐ARHGAP8, and PCDH1. All five genes showed significant case‐control expression differences in both datasets (*p* < 0.001). Given the small APS cohort, the machine‐learning analyses were considered exploratory and were not interpreted as evidence of validated diagnostic performance. CIBERSORT suggested that both diseases have a blood immune profile enriched for myeloid signals, particularly neutrophils and monocyte/macrophage populations, whereas SSc showed relatively stronger CD4+ T‐cell and NK‐cell signals. S100A8 expression was positively correlated with inferred neutrophil abundance in both datasets (APS *r* = 0.62; SSc *r* = 0.58; *p* < 0.05). Finally, miRNA prediction and DSigDB enrichment pointed to possible upstream regulators and candidate compounds, including miR‐155, miR‐146a, celecoxib, tamibarotene, HMN‐176, and XMD14‐99. These findings represent concordant signals observed across two parallel disease cohorts rather than independent cross‐disease validation and therefore require confirmation in independent cohorts.

## 1. Introduction

Autoimmune diseases develop when immune tolerance fails, leading to chronic inflammation and injury across multiple organs. APS is clinically defined by thrombosis and pregnancy morbidity associated with antiphospholipid antibodies [1], whereas SSc is characterized by immune activation, microvascular damage, and progressive fibrosis, as reflected in the 2013 ACR/EULAR classification criteria [2, 3]. Although the clinical manifestations differ, both diseases involve abnormalities in innate and adaptive immunity, cytokine signaling, and endothelial function [4, 5]. This overlap raises the possibility that APS and SSc share blood transcriptional programs that may help explain common pathogenic features and aid biomarker discovery [6, 7].

The rationale for comparing APS and SSc was based on their contrasting manifestations of immune‐mediated vascular pathology rather than on general autoimmune inflammation alone. Although both disorders involve immune activation and vascular injury, their predominant vascular outcomes differ: APS is characterized primarily by antibody‐associated thrombosis, whereas SSc involves persistent microvascular dysfunction followed by vascular remodeling and fibrosis. Comparing these two disorders therefore provides a focused framework for examining whether distinct autoimmune vascular phenotypes share peripheral blood transcriptional programs at the interface of inflammation, immune activation, and vascular injury. Importantly, we do not assume that the APS–SSc pairing is uniquely informative among autoimmune diseases or that the identified signals are specific to these two disorders. Rather, APS and SSc were used as a focused comparative model, and future comparisons with other autoimmune diseases, such as systemic lupus erythematosus and inflammatory myopathies, will be necessary to determine the broader specificity of the identified transcriptional features.

Public databases such as the Gene Expression Omnibus (GEO) provide an opportunity to compare transcriptomic patterns across disease cohorts [8, 9]. Cross‐disease studies of autoimmune disorders have repeatedly identified conserved inflammatory modules, especially interferon‐related signatures, alongside features that remain disease‐specific [10]. At the same time, machine‐learning approaches are being used increasingly to reduce high‐dimensional expression data to smaller sets of genes with potential diagnostic value [11]. Random forests are useful for ranking nonlinear predictors in the presence of correlated variables [12], and neural networks can capture interactions among selected features [13]. Using both methods together may help prioritize candidate features concordantly observed across the two cohorts [14].

Interpretation of whole‐blood transcriptomes is also shaped by changes in immune‐cell composition. Neutrophil extracellular trap formation and monocyte activation have been linked to APS [15], whereas macrophage polarization and T‐cell imbalance are important in SSc [5]. Even so, shared and disease‐specific immune‐cell patterns across APS and SSc have not been examined systematically within a common analytic framework, and the relationship between candidate marker genes and inferred immune‐cell fractions remains incompletely defined. Combining digital cytometry with gene‐level analysis can help distinguish whether bulk transcriptional signals reflect altered cell abundance, altered activation states, or both.

Here, we analyzed peripheral blood transcriptome datasets from APS and SSc as parallel discovery cohorts to identify genes dysregulated in the same direction in both diseases. We then used complementary machine‐learning approaches to prioritize shared candidate genes, estimate immune‐cell composition by digital cytometry, and examine gene–immune associations. Finally, we explored potential upstream miRNAs and drug‐signature enrichment to generate hypotheses for future mechanistic and translational studies [16, 17].

## 2. Materials and Methods

### 2.1. Data Sources and Preprocessing

We downloaded two peripheral blood transcriptome datasets from GEO: GSE102215 for APS (nine patients and nine healthy controls; GPL16791) and GSE231691 for SSc (49 patients and 18 healthy controls; GPL20301). All samples were derived from *Homo sapiens* peripheral blood (Table 1).

**Table 1:** Summary of datasets and available cohort characteristics.

	SSc	APS
GEO accession	GSE231691	GSE102215
Platform	GPL20301	GPL16791
Samples in the disease group	49	9
Samples in the control group	18	9
Species	*Homo sapiens*	*Homo sapiens*
Reference (PMID)	37867940	28931754
Disease/clinical group	Systemic sclerosis/normal controls	Primary APS/healthy controls
Age (years)	Not available	APS: Mean 44.7 (range, 27–57); controls: Mean 43.9 (range, 27–58)
Sex	Not available	APS: Six females/three males; controls: Six females/three males
Reported ethnicity	Not available	APS: Eight White/one Black; controls: Eight White/one Black
Disease duration	Not available	Not available
Treatment status	Not available	Not available

Preprocessing was performed in R (v4.4.1) using standard microarray procedures [18]. Raw data were background‐corrected and normalized with robust multiarray average (RMA). Probe IDs were mapped to official gene symbols using the corresponding platform annotation files, and probes without valid annotation or with ambiguous mapping were removed. When multiple probes mapped to the same gene, their expression values were averaged to generate a single gene‐level measurement.

Because the two datasets came from different studies and different platforms, we did not merge the expression matrices. Instead, all differential expression and feature‐selection analyses were performed separately within each cohort, and shared signals were defined only after comparing concordant results across datasets. This strategy reduced direct platform‐related confounding while preserving the ability to identify common biological patterns [19].

### 2.2. Cohort Demographic and Clinical Characteristics

Available demographic and clinical characteristics were extracted from the sample annotations provided with the public datasets. In GSE102215, the APS cohort comprised nine patients with primary APS and nine healthy controls. The mean ages were 44.7 years (range, 27–57 years) for APS patients and 43.9 years (range, 27–58 years) for controls. Both groups included six females and three males and had identical reported ethnic distributions (eight White and one Black participant in each group). Disease duration and treatment status were not reported in the accessible annotations. In GSE231691, 49 samples were annotated as systemic sclerosis and 18 as normal controls; however, individual‐level information regarding age, sex, disease subtype, disease duration, and treatment status was not available in the public sample annotations. Missing characteristics were reported as unavailable and were not inferred or imputed.

### 2.3. Differential Expression and Shared Gene Definition

Differential expression was analyzed separately in each dataset using edgeR. For the primary exploratory DEG screening, cohort‐specific effect‐size thresholds corresponding to the original analyses were retained: |log2FC| > 1 for APS and |log2FC| > 0.8 for SSc, together with nominal *p* < 0.05. Upregulation and downregulation were defined according to positive and negative logFC values, respectively, among genes meeting the applicable absolute fold‐change threshold. Because the APS cohort was small and the analysis was exploratory, nominal *p* values were used for DEG screening and downstream findings were interpreted cautiously.

Genome‐wide screening necessarily involves a large number of simultaneous tests. For that reason, nominal *p* values from edgeR were treated as exploratory, and priority was given to signals observed with the same direction of change in both datasets. For enrichment analyses, we used the multiple‐testing‐adjusted values provided by clusterProfiler (Benjamini–Hochberg adjustment) and considered adjusted *p* values, or FDR *q* values for GSEA, when assessing significance.

Because the primary differential expression analysis was intended as an exploratory screening step, nominal *p* values were used to define the discovery DEG sets. To assess the robustness of the findings to multiple‐testing correction, we additionally performed a sensitivity analysis using Benjamini–Hochberg FDR‐adjusted *p* values. The original fold‐change thresholds used for each cohort were retained, whereas the significance criterion was changed from nominal *p* < 0.05 to FDR < 0.05. We also examined whether the five prioritized candidate genes remained statistically significant after FDR correction in both cohorts.

Shared differentially expressed genes (shared DEGs) were defined as genes meeting the DEG criteria in both datasets with the same direction of change. Overlap was summarized with Venn diagrams, and volcano plots and heatmaps were used to visualize expression patterns across samples.

### 2.4. Functional Enrichment Analyses

GSEA was carried out separately for each dataset using clusterProfiler and hallmark gene sets (Category H) obtained from msigdbr [18]. Genes were ranked by log2FC, and the results were visualized with enrichplot, including enrichment curves, bubble plots, and ridge plots.

To characterize the shared DEGs, we performed Gene Ontology (GO) and Kyoto Encyclopedia of Genes and Genomes (KEGG) enrichment analyses with clusterProfiler using https://org.hs.eg.db/ for identifier conversion [18]. GO analysis covered biological process, cellular component, and molecular function categories, and KEGG analysis was restricted to *Homo sapiens* pathways (hsa).

### 2.5. Exploratory Machine‐Learning Feature Prioritization

Shared DEGs served as the initial feature pool for exploratory feature prioritization. Random forest classification was performed separately in the APS and SSc cohorts using the randomForest R package. Disease status was treated as a binary outcome (patient = 1, control = 0), and gene‐expression features were used as predictors. Each model used 500 trees (ntree = 500), with 100 variables randomly sampled at each split (mtry = 100) [12]. Variable importance was quantified using the mean decrease in Gini impurity [20]. Genes were ranked by their Gini importance scores, and the Top 30 genes from each cohort were retained. The overlap between the two top‐ranked feature sets was used to identify the five shared candidate genes: S100A8, IER5L‐AS1, LTK, PRR5‐ARHGAP8, and PCDH1. Model performance was internally assessed using the out‐of‐bag (OOB) error rate generated from samples not included in the corresponding bootstrap draw. Given the high dimensionality relative to sample size, particularly in the APS cohort (nine patients and nine controls), these models were used for exploratory feature prioritization rather than to establish validated predictive classifiers.

Artificial neural network (ANN) models were then built with nnet using *z*‐score‐normalized expression values for the shared candidate genes [13]. Each model contained a single hidden layer with three neurons, a maximum of 500 iterations, a learning rate of 0.01, and a fixed random seed of 123. Gene importance was summarized from network weights, and model architecture was visualized with NeuralNetTools. Consistent with the random forest analysis, the ANN models were used only for exploratory feature prioritization and visualization, not for estimating validated predictive performance.

Exploratory ROC analysis was restricted to the larger SSc cohort and performed using pROC [21]. AUC values with 95% confidence intervals were reported descriptively. These within‐cohort estimates were not considered independent validation because feature prioritization and ROC assessment were performed in the same cohort. ROC analysis was not reported for the APS cohort because its small sample size (nine patients and nine controls) precluded reliable estimation of discriminative performance.

### 2.6. Immune Deconvolution and Gene–Immune Association

Relative fractions of 22 immune‐cell types were inferred with CIBERSORT using the LM22 signature matrix [22]. We used 1000 permutations and excluded samples with CIBERSORT output *p* > 0.05.

Differences in immune‐cell fractions between disease and control groups were tested with the Wilcoxon rank‐sum test and displayed with stacked bar plots and boxplots. Associations between candidate‐gene expression and immune‐cell fractions were evaluated with Spearman′s rank correlation [21].

### 2.7. miRNA Prediction and Drug‐Signature Enrichment

Potential upstream miRNAs targeting the prioritized candidate genes were obtained from miRWalk [23]. The resulting miRNA–mRNA interaction network was visualized in Cytoscape [24]. miRNAs previously implicated in immune regulation, such as miR‐155 and miR‐146a, were specifically noted on the basis of prior studies in autoimmune disease [25].

Potential compounds were explored by drug–gene signature enrichment using the Drug Signatures Database (DSigDB) [26]. Because DSigDB links genes to drug‐induced transcriptional signatures, it can highlight compounds whose expression profiles overlap with the genes of interest [27]. Drugs were ranked by enrichment statistics, including adjusted *p* values and combined scores, and the highest‐ranking compounds were treated as hypothesis‐generating candidates.

### 2.8. Software, Statistical Analysis, and Reproducibility

All statistical and bioinformatics analyses were performed using R Version 4.4.1 [18]. Differential expression analysis and expression data processing used edgeR (Version 4.4.2). Functional enrichment analyses used clusterProfiler (Version 4.14.4), msigdbr (Version 24.1.0), enrichplot (Version 1.26.5), and https://org.hs.eg.db/ (Version 3.20.0). Random forest analysis was implemented using randomForest (Version 4.7‐1.2). ANN analysis used nnet (Version 7.3‐19), and network visualization used NeuralNetTools (Version 1.5.3). ROC analyses used ROCR (Version 1.0‐11) and pROC (Version 1.18.5). Data visualization used ggplot2 (Version 4.0.1), pheatmap (Version 1.0.12), and ggpubr (Version 0.6.0). Detailed analytical parameters, including differential expression thresholds, enrichment criteria, random forest settings, neural‐network parameters, CIBERSORT settings, and correlation methods, are provided in the corresponding subsections. All statistical tests were two‐sided, and *p* < 0.05 was considered significant. For analyses involving multiple comparisons, FDR‐adjusted values reported by the corresponding methods were used.

## 3. Results

### 3.1. Study Overview

We first processed the APS and SSc whole‐blood transcriptome datasets separately and then compared the results across cohorts. The analysis workflow included differential expression screening, identification of concordantly dysregulated genes, functional enrichment, exploratory machine‐learning‐based candidate‐feature prioritization, immune‐cell deconvolution, gene–immune correlation analysis, and exploratory miRNA and drug‐signature analyses (Figure 1).

**Figure 1:**
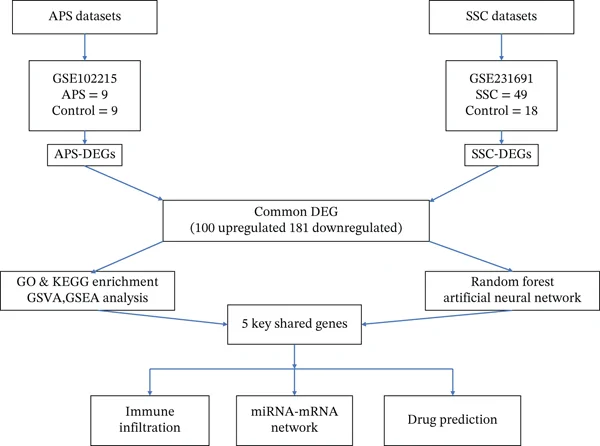
Study design and analytical workflow used to identify concordant signals and immune correlates across the parallel APS and SSc discovery cohorts.

### 3.2. Shared DEGs Across APS and SSc

In the APS cohort, edgeR identified 2744 upregulated and 1058 downregulated genes using |log2FC| > 1 and nominal *p* < 0.05, giving 3802 DEGs overall. In the SSc cohort, edgeR identified 361 upregulated and 1566 downregulated genes using |log2FC| > 0.8 and nominal *p* < 0.05, giving 1927 DEGs overall. When we intersected genes that changed in the same direction in both datasets, 281 shared DEGs remained, including 100 commonly upregulated and 181 commonly downregulated genes (Figure 2; Supporting Information 1: Table S1 and Supporting Information 2: Table S2). The larger number of DEGs in APS likely reflects the small sample size, inter‐individual variability, and the use of nominal *p* values without additional filtering [28, 29].

**Figure 2:**
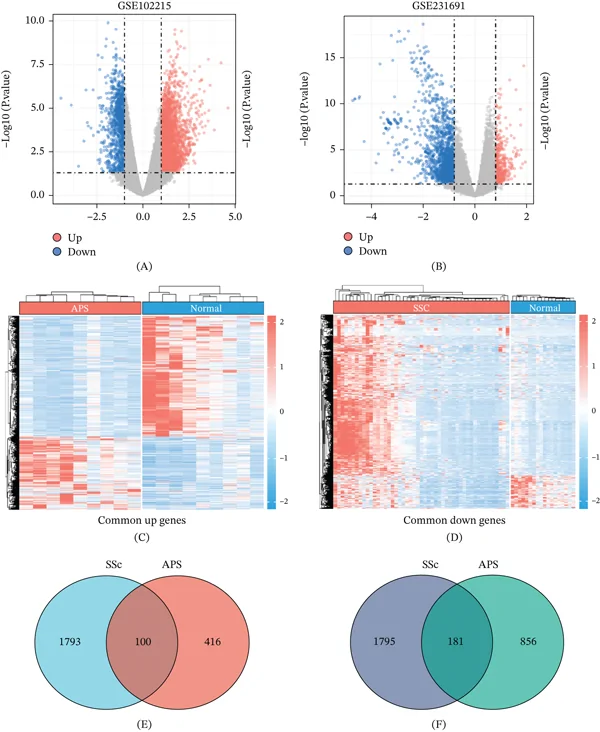
Differential expression and shared DEGs in APS and SSc. Volcano plots, expression heatmaps, and clustering summarize differential expression in each cohort and support the 281 concordantly regulated shared DEGs (100 up and 181 down). APS: (A) volcano plot, (C) DEG heatmap, and (E) hierarchical clustering with group annotation. SSc: (B) volcano plot, (D) DEG heatmap, and (F) hierarchical clustering with group annotation.

As a sensitivity analysis, we repeated DEG filtering using FDR‐adjusted significance criteria while retaining the original cohort‐specific fold‐change thresholds. In the APS cohort, 3190 DEGs remained after FDR correction, including 2233 upregulated and 957 downregulated genes, compared with 3802 DEGs identified using nominal *p* values. In the SSc cohort, 1656 DEGs remained after FDR correction, including 295 upregulated and 1361 downregulated genes, compared with 1927 DEGs in the nominal‐P analysis. Thus, a substantial proportion of the DEG signals identified in the primary analysis remained significant after multiple‐testing correction. Importantly, all five prioritized candidate genes—S100A8, IER5L‐AS1, LTK, PRR5‐ARHGAP8, and PCDH1—remained statistically significant at FDR < 0.05 in both cohorts, supporting the robustness of these candidate‐level signals.

The graphical summaries were consistent with these overlap results. Volcano plots (Figure 2A for APS and Figure 2B for SSc) show the case‐control expression changes in each cohort. Heatmaps of the shared DEGs (Figure 2C,D) and hierarchical clustering (Figure 2E,F) further indicate that this shared gene set captures clear differences between disease and control samples in both datasets.

### 3.3. Pathway Enrichment Reveals Convergent Immune and Inflammatory Programs

Hallmark GSEA showed that immune and inflammatory pathways were prominent in both diseases. In APS, interferon‐alpha and interferon‐gamma response signatures were among the strongest findings, together with other cytokine‐ and inflammation‐related programs (Figure 3A,B). SSc displayed a broadly similar enrichment pattern (Figure 3C,D). These results are consistent with previous work implicating interferon and cytokine dysregulation in both APS and SSc [5, 15].

**Figure 3:**
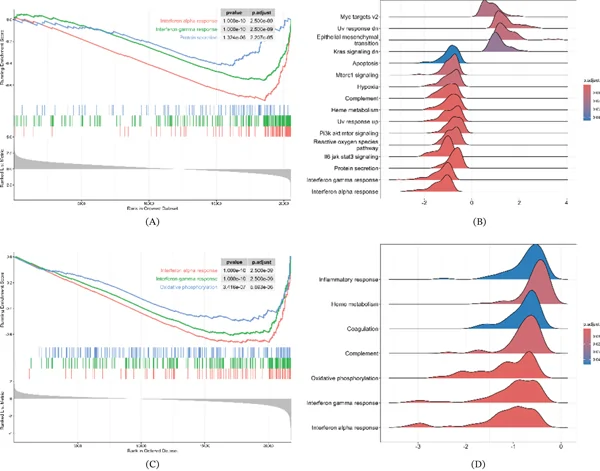
Hallmark GSEA in APS and SSc. GSEA highlights immune/inflammatory programs shared across diseases. APS: (A) enrichment curves for representative hallmark sets and (B) ridge plot summarizing NES across hallmark sets (colored by FDR). SSc: (C) enrichment curves for representative hallmark sets and (D) ridge plot summarizing NES across hallmark sets (colored by FDR).

GO and KEGG analyses of the shared DEGs supported the same general picture (Figure 4). Enriched GO terms were dominated by immune‐response and host‐defense processes (Figure 4A,B), whereas KEGG analysis highlighted pathways including TNF and NF‐*κ*B signaling (Figure 4C,D), both of which are relevant to chronic inflammatory responses [30].

**Figure 4:**
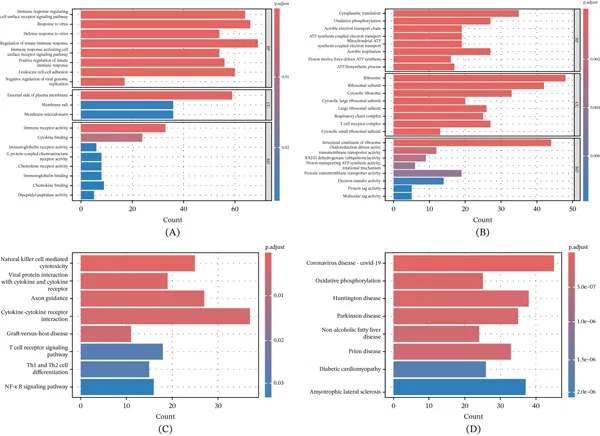
GO and KEGG enrichment of shared DEGs. GO and KEGG analyses of shared DEGs implicate immune and inflammatory functions. GO bar plots: (A) APS and (B) SSc, grouped by BP/CC/MF. KEGG bar plots: (C) APS and (D) SSc, highlighting significantly enriched pathways.

### 3.4. Exploratory Machine Learning Prioritizes Five Shared Candidate Genes

Random forest analyses performed separately in the two cohorts ranked genes according to feature importance. Intersecting the Top 30 genes from SSc (Figure 5A) and APS (Figure 5B) identified five shared high‐importance candidates: S100A8, IER5L‐AS1, LTK, PRR5‐ARHGAP8, and PCDH1 (Figure 5C) [11, 13]. These genes were consistently prioritized in both disease cohorts and were retained as shared candidate features for subsequent exploratory analyses. The OOB error rates of the random forest classifiers were 11.11% in the APS cohort and 10.45% in the SSc cohort. Given the limited sample size, particularly in APS, these estimates were treated as internal model diagnostics rather than evidence of independently validated predictive performance.

**Figure 5:**
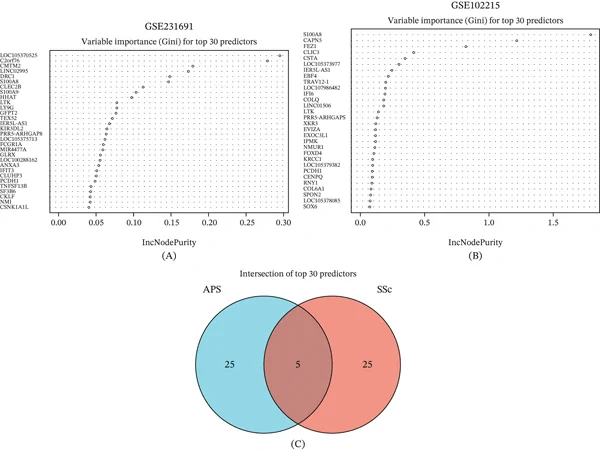
Random forest feature ranking and overlap across cohorts. Random forest importance ranks the highest importance genes in (A) SSc and (B) APS (IncNodePurity). (C) Venn overlap of the Top 30 genes identifies five shared high‐importance candidates: S100A8, IER5L‐AS1, LTK, PRR5‐ARHGAP8, and PCDH1.

In the larger SSc cohort, exploratory ROC analysis indicated that the five candidate genes showed varying degrees of within‐cohort separation between cases and controls (Figure 6A–E). The AUC estimates are presented descriptively and should not be interpreted as independently validated diagnostic performance. ROC results were not reported for APS because of the limited cohort size.

**Figure 6:**
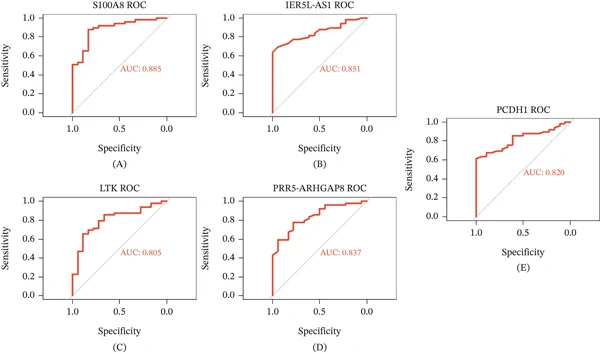
Exploratory ROC analysis of the five candidate genes in the SSc cohort. ROC curves for (A) S100A8, (B) IER5L‐AS1, (C) LTK, (D) PRR5‐ARHGAP8, and (E) PCDH1 in the SSc cohort. AUC values and 95% confidence intervals are shown descriptively. Because feature selection and ROC assessment were performed within the same cohort, these estimates do not constitute independent validation of diagnostic performance. APS ROC curves and AUC estimates are not reported because of the limited APS cohort size.

ANN models built from the five‐gene panel were used to explore how the shared candidate features captured disease‐related expression patterns. The APS and SSc network structures are shown in Figure 7A,B. Boxplots of *z*‐score‐normalized expression (Figure 7C,D) show significant case‐control differences for all five genes (Wilcoxon′s rank‐sum test, *p* < 0.001), and the direction of change was concordant across the two disease cohorts.

**Figure 7:**
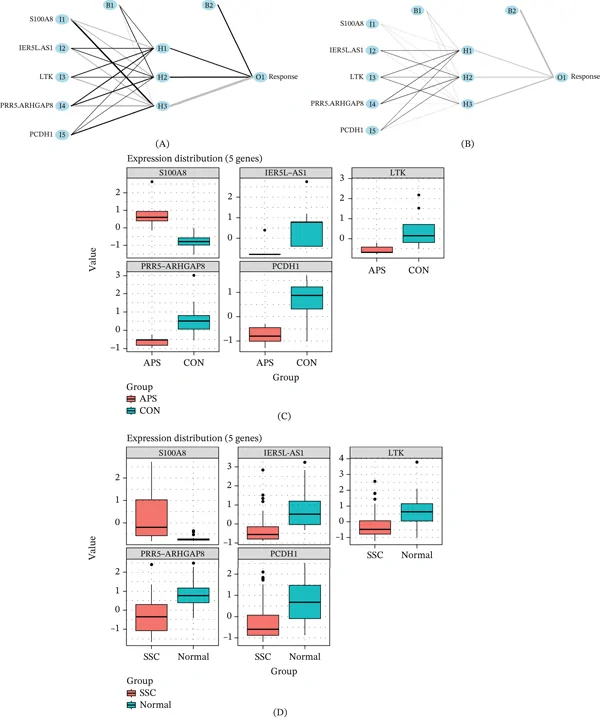
Exploratory ANN modeling and case‐control expression patterns of five shared candidate genes. ANN models using the five genes as inputs in (A) APS and (B) SSc (single hidden layer with three neurons; max 500 iterations; learning rate 0.01; seed 123). Boxplots show *z*‐score–normalized expression differences between cases and controls in (C) APS and (D) SSc (Wilcoxon′s rank‐sum test, *p* < 0.001).

### 3.5. Immune Infiltration Patterns and Gene–Immune Associations

CIBERSORT deconvolution suggested that both APS and SSc share a predominantly myeloid blood signature. Immune‐cell fraction heatmaps (Figure 8A for APS and Figure 8C for SSc) showed stronger myeloid‐associated signals, and clustering based on inferred immune‐cell composition separated cases from controls in both cohorts (Figure 8B,D) [22]. In SSc, inferred CD4+ T‐cell and NK‐cell signals were more evident than in APS (Figure 8C), which is in keeping with the broader adaptive immune involvement described in SSc [4, 5].

**Figure 8:**
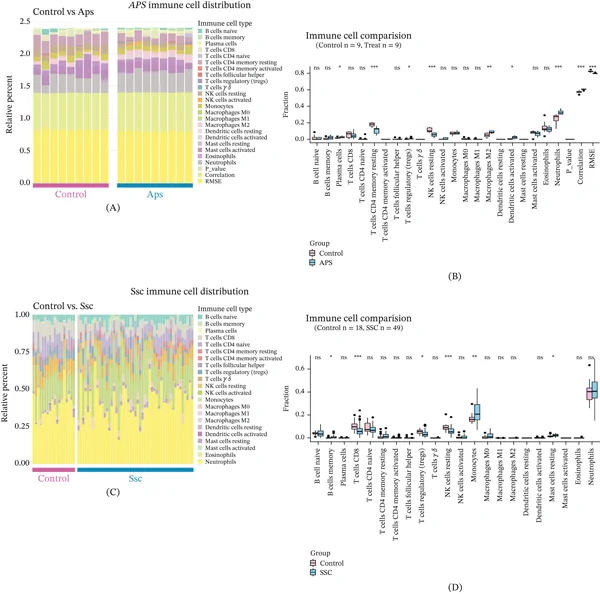
Immune‐cell infiltration patterns in APS and SSc. CIBERSORT‐estimated immune‐cell fractions across samples. APS: (A) heatmap of 22 immune‐cell types by sample and (B) clustering with group annotation. SSc: (C) heatmap and (D) clustering with group annotation. Statistical significance was indicated as follows: ns, not significant; *p < 0.05; **p < 0.01; ***p < 0.001.

Correlation analysis connected the selected candidate genes with specific immune‐cell fractions (Figure 9). S100A8 expression was positively associated with inferred neutrophil abundance in both datasets (APS *r* = 0.62; SSc *r* = 0.58; *p* < 0.05), consistent with its myeloid‐biased expression and proinflammatory role [30, 31]. PRR5‐ARHGAP8 and PCDH1 were also strongly correlated with resting NK cells across both diseases (|*r*| ≥ 0.6; *p* < 0.001), suggesting that the five‐gene panel captures variation in both myeloid and lymphoid compartments (Figure 9; Supporting Information 3: Table S3 and Supporting Information 4: Table S4).

**Figure 9:**
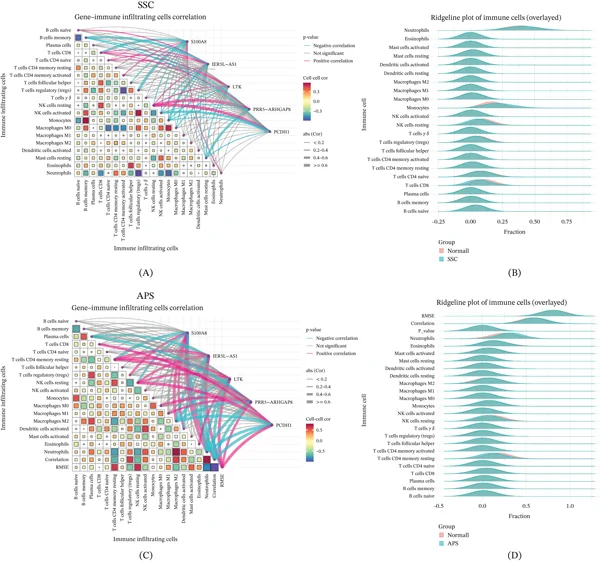
Gene–immune associations in APS and SSc. Spearman′s associations between five shared candidate genes and inferred immune‐cell fractions. SSc: (A) gene–cell correlation network and (B) ridgeline plot of immune‐cell fractions. APS: (C) gene–cell correlation network and (D) ridgeline plot of immune‐cell fractions.

### 3.6. miRNA Network and Candidate Drug Prediction

miRWalk analysis identified several immune‐related miRNAs as potential upstream regulators of the shared candidate genes [23]. In the resulting network, miR‐155‐5p and miR‐146a‐5p appeared as multitarget nodes (Figure 10; Supporting Information 5: Table S5), consistent with their established roles in inflammatory feedback and innate immune signaling [24, 25].

**Figure 10:**
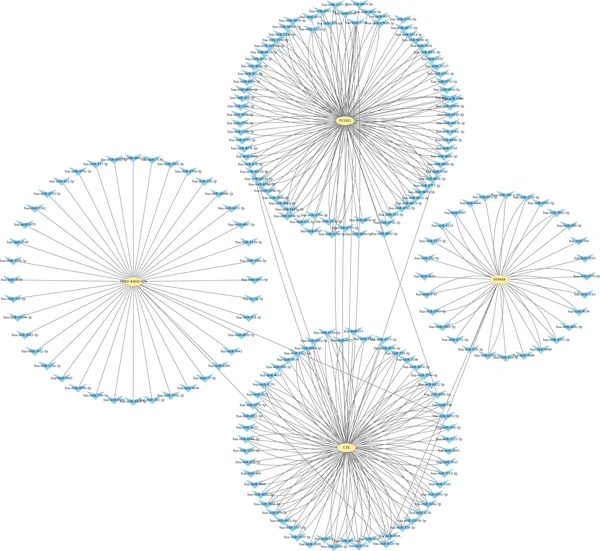
Predicted miRNA–mRNA regulatory network for shared candidate genes. miRWalk‐predicted miRNAs targeting S100A8, LTK, PRR5‐ARHGAP8, and PCDH1 are shown; IER5L‐AS1 had no miRWalk targets in this analysis. Circles denote miRNAs, and squares denote mRNAs; edges indicate predicted interactions. Notable multitarget miRNAs include miR‐155‐5p (targets S100A8 and LTK) and miR‐146a‐5p (targets S100A8 and PCDH1).

DSigDB enrichment identified several compounds whose transcriptional signatures overlapped with the shared candidate genes, including celecoxib, tamibarotene, HMN‐176, and XMD14‐99 (Table 2; Supporting Information 6: Table S6) [26]. These findings are intended as leads for follow‐up work rather than evidence of therapeutic efficacy in APS or SSc.

**Table 2:** Top candidate compounds from DSigDB enrichment analysis.

Drug names	*p* value	Adjusted *p* value	Odds ratio	Combined score	Genes
XMD14‐99 LINCS	0.0057	0.0717	226.96	1171.31	LTK
Celecoxib MCF7	0.0059	0.0717	217.08	1111.11	S100A8
Tamibarotene CTD	0.0073	0.0717	23.26	114.2303167	PCDH1; S100A8
HMN‐176 CTD	0.0077	0.0717	166.37	809.09	PCDH1

## 4. Discussion

### 4.1. Principal Findings

This study identified a shared blood transcriptional signature across APS and SSc and prioritized five candidate genes using two complementary machine‐learning approaches. The 281 shared DEGs were enriched in immune and inflammatory pathways, particularly interferon‐related programs, which fits with prior reports of conserved transcriptional modules across autoimmune diseases [10]. The five prioritized genes—S100A8, IER5L‐AS1, LTK, PRR5‐ARHGAP8, and PCDH1—were identified through concordant exploratory feature‐prioritization analyses across the two cohorts and showed consistent case‐control expression differences. Given the limited sample size, particularly for APS, these findings should be regarded as exploratory rather than as evidence of validated diagnostic performance. Immune deconvolution further suggested that both diseases are characterized by a myeloid‐skewed blood profile [22], whereas SSc showed relatively stronger adaptive immune signals, in line with its clinical and immunologic heterogeneity [4, 5].

Importantly, the concordant signals observed in APS and SSc should not be interpreted as cross‐disease replication or independent validation. The two datasets represent parallel discovery cohorts from different diseases, and their overlap indicates that certain transcriptional features are shared between APS and SSc. Independent replication will require additional APS and SSc cohorts that were not involved in the discovery process.

### 4.2. Biological Interpretation of Prioritized Candidate Genes

S100A8 encodes a calcium‐binding protein that is abundant in neutrophils and monocytes and is known to amplify inflammatory responses [31]. Its consistent differential expression in both cohorts, together with the positive correlation with inferred neutrophil abundance, supports the idea that a shared myeloid‐inflammatory axis contributes to both APS and SSc [30]. Previous studies have also linked the S100A8/A9 complex to Th17‐related responses in autoimmune disease [30].

IER5L‐AS1 is a long noncoding RNA with little direct functional evidence in APS or SSc. However, lncRNAs can regulate immune processes and are increasingly implicated in autoimmune pathogenesis [30]. Its concordant dysregulation across the two disease cohorts makes it an interesting candidate, although its cellular source and downstream targets still need to be defined experimentally.

LTK is a receptor tyrosine kinase involved in cell signaling [32]. PRR5‐ARHGAP8 has links to Rho GTPase‐related pathways, and PCDH1 participates in cell–cell adhesion and barrier‐related functions [33]. At present, the roles of these genes in APS or SSc remain speculative, but they may connect immune activation with endothelial dysfunction, tissue remodeling, or fibrotic responses. This possibility will need direct experimental testing.

### 4.3. Shared and Disease‐Specific Immune Landscapes

The immune deconvolution results showed both overlap and distinction between the two diseases. In both cohorts, higher neutrophil and monocyte/macrophage signals were consistent with chronic inflammation and vascular involvement [15, 22]. In APS, neutrophil extracellular traps have been implicated in thrombosis [15], whereas in SSc, macrophage polarization and T‐cell imbalance are linked to fibrotic remodeling [5]. The relatively stronger CD4+ T‐cell and NK‐cell signals in SSc fit with broader adaptive immune dysregulation in that disease [5]. Taken together, the gene–immune correlations suggest that the candidate‐gene signals are influenced by immune‐cell composition and activation state rather than by cell‐intrinsic transcriptional changes alone.

### 4.4. Regulatory and Translational Hypotheses

miRNA analysis pointed to regulatory candidates that may act on more than one shared candidate gene [23]. In light of the known roles of miR‐155 and miR‐146a in inflammatory feedback and innate immune signaling [24, 25], these interactions provide concrete hypotheses for follow‐up studies on post‐transcriptional regulation of the shared signature. For example, inhibition of miR‐155 has been reported to dampen inflammation in autoimmune models [25], raising the possibility that it may influence part of the shared program observed here.

Drug‐signature enrichment highlighted celecoxib, tamibarotene, HMN‐176, and XMD14‐99 [26]. These hits simply indicate overlap between drug‐associated transcriptional signatures and the genes identified in this study; they should not be interpreted as evidence that these agents are effective in APS or SSc. Their main value at this stage is to provide a starting point for experimental screening or mechanistic follow‐up [27].

### 4.5. Limitations and Future Directions

Several limitations should be noted. First, the APS cohort was very small (nine patients and nine controls), whereas the number of candidate molecular features greatly exceeded the number of samples. This high‐dimensional, small‐sample setting creates a substantial risk of overfitting and precludes reliable estimation of predictive performance. Accordingly, the random forest and ANN analyses should be interpreted as exploratory feature‐prioritization procedures rather than validated predictive models. We therefore did not report APS ROC/AUC estimates. Confirmation of the five candidate genes in substantially larger, independent APS and SSc cohorts not involved in the discovery process is required before their diagnostic utility can be established. Second, the two datasets were generated on different microarray platforms; although we avoided direct data merging and focused on concordant within‐dataset findings, some platform‐related effects may remain [19]. Third, this was a computational study based entirely on public data, so independent cohort validation and mechanistic experiments are still needed [13]. Fourth, CIBERSORT provides relative estimates derived from bulk expression profiles [22], and orthogonal approaches such as flow cytometry or single‐cell analysis would strengthen these inferences [17]. Finally, the DSigDB results are exploratory and require experimental evaluation [27].

Although the primary DEG discovery used nominal *p* values to preserve sensitivity in the exploratory analysis, the additional FDR‐based sensitivity analysis showed that a substantial proportion of DEG signals remained significant in both cohorts, and all five prioritized candidate genes retained FDR‐adjusted significance. Nevertheless, the nominal‐P discovery strategy may still increase the false‐positive rate, and the shared‐gene findings should therefore be interpreted as exploratory pending confirmation in independent cohorts.

Several potential confounding factors should also be considered. Although the APS cases and controls showed comparable distributions of age, sex, and reported ethnicity, detailed clinical information was not uniformly available across the two public cohorts. In particular, individual‐level demographic characteristics were unavailable for the SSc cohort, and information regarding disease duration and treatment status was unavailable or incomplete. Consequently, the potential effects of age, sex, disease duration, treatment exposure, disease subtype, and other unmeasured clinical factors on gene‐expression patterns could not be formally adjusted for. These factors may contribute to inter‐individual transcriptional variability and residual confounding. Therefore, the shared molecular signals identified in this study should be interpreted as exploratory, and future confirmation in larger, clinically well‐characterized cohorts with comprehensive demographic and treatment information is warranted.

Future studies should test the five‐gene panel in larger, multicenter cohorts and examine the cellular origin of the shared signals using single‐cell approaches [16]. Mechanistic work focused on myeloid‐inflammatory pathways, particularly those involving S100A8, may help clarify how this shared blood signature relates to immune dysregulation and vascular injury in APS and SSc [31].

**Nomenclature**
ACR: American College of Rheumatology
ANN: artificial neural network
APS: antiphospholipid syndrome
AUC: area under the curve
CIBERSORT: cell‐type identification by estimating relative subsets of RNA transcripts algorithm
DEG: differentially expressed gene
DEGs: differentially expressed genes
DSigDB: Drug Signatures Database
EULAR: European Alliance of Associations for Rheumatology
FDR: false discovery rate
GEO: Gene Expression Omnibus
GO: Gene Ontology
GSEA: gene set enrichment analysis
KEGG: Kyoto Encyclopedia of Genes and Genomes
LM22: leukocyte gene signature matrix of 22 immune‐cell types
lncRNA: long noncoding RNA
miRNA: microRNA
mRNA: messenger RNA
NF‐*κ*B: nuclear factor kappa‐light‐chain‐enhancer of activated B cells
NK: natural killer (cells)
OOB: out‐of‐bag
RMA: robust multiarray average
ROC: receiver operating characteristic
SSc: systemic sclerosis
TNF: tumor necrosis factor

## Author Contributions

Renju Yang and Jinchan Liu have contributed to the work equally and should be regarded as cofirst authors.

## Funding

No funding was received for this manuscript.

## Disclosure

An earlier version of this manuscript has previously been made publicly available as a preprint. For transparency, the preprint has been cited in the reference list.

## Ethics Statement

All data analyzed in this study were obtained from public GEO repositories. The original studies reported ethical approval and informed consent. Our work consisted only of secondary analysis of publicly available deidentified data and did not involve new experiments in human participants.

## Conflicts of Interest

The authors declare no conflicts of interest.

## Supporting Information

Additional supporting information can be found online in the Supporting Information section.

## Supporting information


**Supporting Information 1** Table S1: Differentially expressed genes identified in the APS cohort (GSE102215) using the predefined screening criteria.

**Supporting Information 2** Table S2: Differentially expressed genes identified in the SSc cohort (GSE231691) using the predefined screening criteria.

**Supporting Information 3** Table S3: Spearman′s correlation results between the five shared candidate genes and inferred immune‐cell fractions in the APS cohort.

**Supporting Information 4** Table S4: Spearman′s correlation results between the five shared candidate genes and inferred immune‐cell fractions in the SSc cohort.

**Supporting Information 5** Table S5: Predicted miRNA–mRNA interactions for the shared candidate genes generated using miRWalk.

**Supporting Information 6** Table S6: Full DSigDB enrichment results for candidate compounds associated with the shared candidate genes.

## Data Availability

All transcriptome datasets analyzed here are available in GEO under Accession Numbers GSE102215 and GSE231691. The analysis code is available from the corresponding author upon reasonable request.
